# Predicting residential structures from open source remotely enumerated data using machine learning

**DOI:** 10.1371/journal.pone.0204399

**Published:** 2018-09-21

**Authors:** Hugh J. W. Sturrock, Katelyn Woolheater, Adam F. Bennett, Ricardo Andrade-Pacheco, Alemayehu Midekisa

**Affiliations:** 1 Global Health Group, University of California, San Francisco, CA, United States of America; 2 Clinton Health Access Initiative, Boston, MA, United States of America; UCLA, UNITED STATES

## Abstract

Having accurate maps depicting the locations of residential buildings across a region benefits a range of sectors. This is particularly true for public health programs focused on delivering services at the household level, such as indoor residual spraying with insecticide to help prevent malaria. While open source data from OpenStreetMap (OSM) depicting the locations and shapes of buildings is rapidly improving in terms of quality and completeness globally, even in settings where all buildings have been mapped, information on whether these buildings are residential, commercial or another type is often only available for a small subset. Using OSM building data from Botswana and Swaziland, we identified buildings for which ‘type’ was indicated, generated via on the ground observations, and classified these into two classes, “sprayable” and “not-sprayable”. Ensemble machine learning, using building characteristics such as size, shape and proximity to neighbouring features, was then used to form a model to predict which of these 2 classes every building in these two countries fell into. Results show that an ensemble machine learning approach performed marginally, but statistically, better than the best individual model and that using this ensemble model we were able to correctly classify >86% (using independent test data) of structures correctly as sprayable and not-sprayable across both countries.

## Introduction

Having accurate maps depicting the locations of buildings across a region benefits a range of sectors. For example, having information on the locations of residential buildings is potentially useful for planning and directing public health activities such as vaccination campaigns and other household interventions. This information can also be used to better estimate population densities and distribution. Despite the availability of detailed data in well-resourced countries via platforms such as Google maps and OpenStreetMap, and numerous efforts to identify buildings from high resolution satellite imagery [1–3], building data are patchy and often completely absent in developing countries.

OpenStreetMap, via the Humanitarian OpenStreetMap (www.hotosm.org) and Missing Maps projects (www.missingmaps.org), is helping to overcome this data gap. Relying mainly on volunteers to search through imagery and manually enumerate maps by tracing their footprints, OSM is building and curating increasingly complete and detailed maps of infrastructure. For example, every building in malaria endemic areas (>600,000 buildings) of the Kingdom of Swaziland has been mapped and efforts are ongoing in Zambia, Zimbabwe and other neighbouring countries.

One driver of demand for these data is the malaria community, who are using OSM and other remotely enumerated building data to help plan and guide intervention campaigns, such as indoor residual spraying (IRS) with insecticide, distribution of insecticide treated nets, and mass drug administration [4, 5]. Data on structures are also useful for foci investigation, community health worker allocation and development of sampling frames for surveys. As household interventions such as IRS are essentially focused only on residential structures, having accurate information on which structures in an area are sprayable is highly valuable. Such information allows programs to better estimate needs for human, financial and physical (vehicles, type and amount of insecticide, spray equipment etc.) resources and can be used to optimize the distribution, size and deployment of spray teams. However, despite the growing availability of data on structures in malaria endemic countries, information pertaining to their use, such as whether a building is commercial, residential or public, is often incomplete.

While on the ground efforts to classify OSM building data, such as those led by the Peace Corps, are helping to address this issue, only a subset of buildings in any given country typically have this information, limiting the usefulness of these data for planning IRS operations. Studies using very high resolution remotely sensed data have shown that it is possible to classify buildings by type using various machine learning methods [6, 7], however, the reliance on very high resolution commercial imagery restricts the applicability of this approach to large scales and resource limited settings.

As others have noted, it may be possible to differentiate residential and non-residential buildings from characteristics such as size and shape [5], which can be calculated from OSM data. Similarly, it is conceivable that proximity to neighbouring buildings and roads may also be related to whether a building is residential or not. To test whether building type can be predicted using open data, here we describe the development and evaluation of a classification algorithm. Rather than relying on a single classification method, such as logistic regression or random forest, we make use of ensemble methods in an effort to boost predictive capacity. We hypothesize that building type can be reliably predicted from freely available predictors and that an ensemble approach provides noticeably superior predictive performance to the best performing base learner.

## Methods

### OSM data

OSM building (polygon) and road (line) data for Botswana and Swaziland were extracted in June 2017 from the third party site Geofabrik which is updated weekly (www.geofabrik.de). These data are generated and validated by the OSM community who are essentially comprised of volunteers around the world. Building footprints and roads, as well as other landscape features such as rivers, are traced manually from high resolution remotely sensed imagery and tagged with key characteristics. Where possible, on the ground efforts are used to augment these data and to provide additional information such as building or road type.

For each structure, the following attributes were generated to be used as predictors in the modeling process. Area, number of sides, number of sub-polygons (courtyards etc.), distance to nearest road, distance to neighbouring structure, area of nearest neighbouring structure, number of sides of nearest neighboring structure and number of sub-polygons of nearest neighboring structure. These attributes were calculated by importing the building and road data into R and using functions from the sp package. Also, using an open access 30m resolution raster layer depicting the probability of being man-made impervious surface (metal, tile, asphalt, concrete) generated by a parallel study [8], we also extracted the impervious value of each structure and its nearest neighbor using the raster package. In addition to the impervious value for the 30m pixel in which the structure lay, we also calculated a local impervious value for the 9x9 (270m x 270m) square buffer around the structure. This was done in an effort to determine the urbanicity of the local area. As there was evidence that structure type data was spatially clustered, i.e. there were clusters of efforts to type commercial or residential structures, latitude and longitude were not included as predictors as it could have led to spurious predictions. For example, if a discrete project focused on tagging commercial structures took place in a particular area, including latitude and longitude in a model would incorrectly classify all structures in that area as commercial. While this may lead to high performance when using cross validation, erroneous results would be produced if new prediction data in that area include residential structures.

For modeling purposes, for each country data were restricted to structures with a value in the ‘type’ field used to describe the function of the building (http://wiki.openstreetmap.org/wiki/Key:building). For the purpose of this study, structure type was reduced to a binary classification of “sprayable” and “not-sprayable”. Table 1 shows a complete list of the structure types found in Botswana and Swaziland combined and their respective classification.

**Table 1:** Structure types of OSM buildings for Swaziland and Botswana combined and their respective binary classification for modeling.

Classification	Type
Sprayable	Abandoned hut, Apartments, Bridge, Building, Cathedral, Church Civic, Collapsed, College, Commercial, Compound, Construction, Constructions, Damaged, Farm, Farm auxiliary, Garage, Government building, Greenhouse, Hangar, Hospital, Hotel, Industrial, Kindergarten, Land Board, Mosque, Office, Public, Quay, Retail, Roof, Roofless, Ruins, School, Service, Shed, Sports centre, Stable, Stadium, Storage tank, Supermarket, Terrace, Toilets, Tree, Tribal Government Building, University, Warehouse, Water utility, Youth ministry condo
Not-sprayable	Detached, House, Hut, Residential, Teacher housing

For model selection purposes, 90% of sprayable and not-sprayable structures were randomly selected to act as training data, with the remaining data acting as a test data. Test data were used to estimate the predictive accuracy (generalization error) of the final model.

### Modeling

To model whether a structure was sprayable or not, we employed a stacked generalization approach [9]. Rather than relying on a single modeling approach such as a logistic regression or a decision tree, stacked generalization or stacking is an approach to applying several modeling approaches (hereby termed base learners) in parallel and combining outputs as a means to reduce the statistical variance, overcome computational problems or expand the representational capacity of the base learners. Thus the likelihood of poor performance can eventually be reduced [10–12]. This is in contrast to bagging or boosting, which relies on replicates of a same base learner [13]. Stacking has been applied to a range of problems from classification to prediction of continuous outcomes and spatial processes [14–16].

In this study, we used the super learner stacking ensemble which, using cross validation as a selection criteria, has been shown to perform at least as well as the best performing base learner [14]. Here, the following base learners were chosen: logistic regression, multivariate adaptive polynomial splines [17], random forest [18], generalized boosted regression [19], elastic net regression [20], conditional inference forest [21], extreme gradient boosting [19] and generalized additive model [22]. Base learners were chosen to represent a range of different modeling approaches and were fit to the training data using the SuperLearner package in R. Default tuning parameters were used.

To ensemble the base learners in order to maximize predictive performance, we used the super learner algorithm that identifies the optimal convex combination of weights to apply to the base learners to maximise 10-fold cross-validated area under the receiver operator curve (AUC) values. AUC values correspond to the probability that a randomly selected truly sprayable structure will have a higher prediction value (i.e. predicted probability or fraction of votes) from the model than a randomly selected truly not-sprayable structure. This super learner algorithm was employed as the primary focus of the study was to maximize the ability to differentiate sprayable from non-sprayable structures (i.e. maximize AUC).

To evaluate the performance of each base learner as well as the ensemble model, and to choose a final modeling approach, we compared the 10-fold cross validated AUC values (CV-AUC). DeLong’s test was used to compare CV-AUC values of the best performing base learner to the super learner [23].

To identify the optimal cutoff to convert predicted probabilities of the final model to hard classifications of 0 (not-sprayable) and 1 (sprayable), we identified the cutoff that provided equally good performance predicting sprayable and not-sprayable structures. To do this, using the cross validated predictions, we plotted the percentage of sprayable (sensitivity) and not-sprayable (specificity) structures correctly classified across all prediction cutoff values and identified the cutoff at which performance was equal.

To test the predictive accuracy of the final model, we predicted the probability of being sprayable using the test data which in turn was used to calculate AUC values. To evaluate whether performance of the model varied by level of urbanicity, we also compared AUC values across quantiles of local impervious values. Using the optimal cutoff identified during model building phase, test data predictions were also converted to classifications (sprayable and not-sprayable) and used to generate a confusion matrix.

To predict the total number of residential and non-residential structures in each country’s dataset, the super learner model was used to predict structure type for every structure in the country for which no ‘type’ value was available. These predictions were then classified using the optimal cutoff identified during model building phase.

## Results

**Fig 1:**
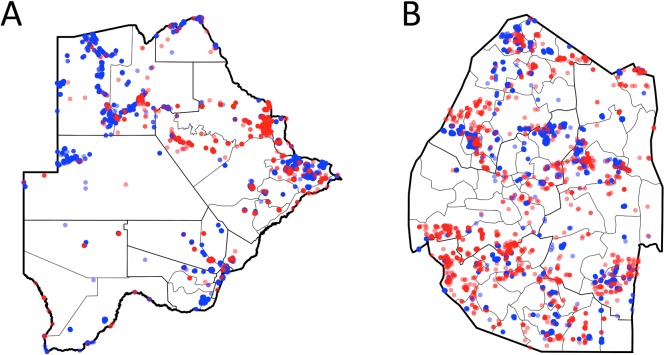
**Distribution of OSM structure data for which type is indicated in A- Botswana and B—Swaziland**. Blue and red points represent sprayable and not-sprayable respectively. Note that data are plotted with opaque colors to allow density to be represented. © OpenStreetMap contributors.

There were a total of 39,174 (out of 610,278, or 6.4%) and 9,177 (out of 593,365, or 1.5%) OSM structures with data on structure type in Botswana and Swaziland respectively. Table 2 shows the breakdown of these data and Fig 1 shows the distribution of the data across the two countries. Data on both sprayable and not-sprayable structures were available from throughout both countries.

**Table 2:** Breakdown of details of the OSM structure data available for Swaziland and Botswana.

Country		Total with ‘type’ data	Total without ‘type’ data
Botswana		39,174	571,104
	Sprayable	33,955	
	Not-sprayable	5,219	
Swaziland		9,177	584,188
	Sprayable	7,317	
	Not-sprayable	1,860	

**Fig 2:**
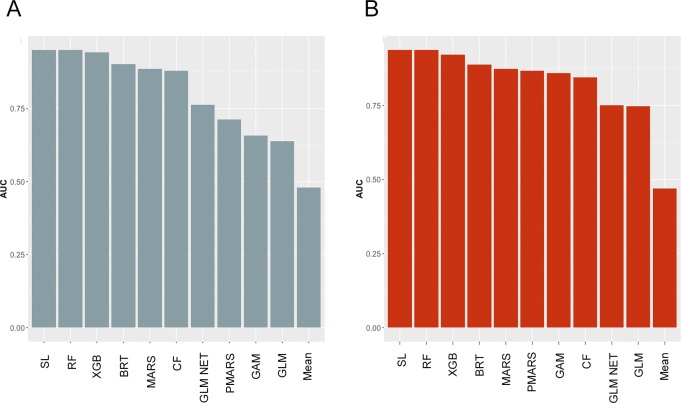
**CV-AUC values obtained from each of the base learners and super learner plotted in decreasing value of AUC for A- Botswana and B–Swaziland.** SL–super learner, RF–random forest, XGB–extreme gradient boosting, BRT–boosted regression tree, MARS–multivariate adaptive regression spline, CF–conditional forest, GLM NET–elastic net regression, PMARS–multivariate adaptive polynomial spline, GLM–logistic regression, GAM–generalized additive model.

Fig 2. shows the CV-AUC values obtained from base learners as well as the super learner and Table 3 shows the coefficients for each base learner estimated by the super learner algorithm. In both countries, random forest was the best performing base learner, with CV-AUC values nearly identical to the super learner (0.950 v 0.951 and 0.937 v 0.938 in Botswana and Swaziland respectively). Boosted regression tress, extreme boosting machines and multivariate adaptive regression splines also performed well in both settings achieving CV-AUC values of 0.868–0.942. DeLong’s test revealed that in both settings, super learner AUC values were significantly higher than random forest (p<0.001 and p = 0.036 in Botswana and Swaziland respectively).

**Table 3:** Coefficients estimated for each base learner by the super leaner algorithm.

Base learner	Botswana	Swaziland
Mean	0.12	0.14
GLM	0	0
PMARS	0.04	0
GLM NET	0	0
BRT	0	0
XGB	0.2	0.26
CF	0.02	0
RF	0.60	0.70
MARS	0	0
GAM	0.01	0

GLM–logistic regression, PMARS–multivariate adaptive polynomial spline, GLM NET–elastic net regression, BRT–boosted regression tree, XGB–extreme gradient boosting, CF–conditional inference forest, RF–random forest, MARS–multivariate adaptive regression spline, GAM–generalized additive model

**Fig 3:**
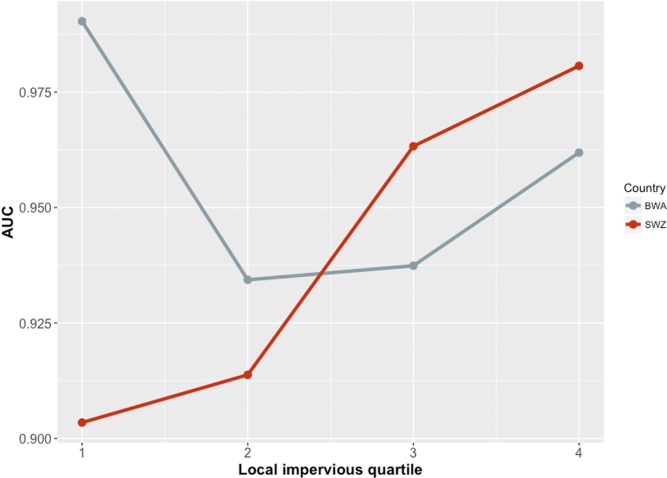
AUC values of the final super learner model by local impervious (urbanicity) quantile for Botswana and Swaziland.

Fig 3. **Performance of the final super learner model on the test data as a function of level of local impervious (urbanicity) quantile.** In Swaziland, AUC increased with increasing level of urbanicity, whereas in Botswana, AUC values were highest in very rural and very urban areas.

**Fig 4:**
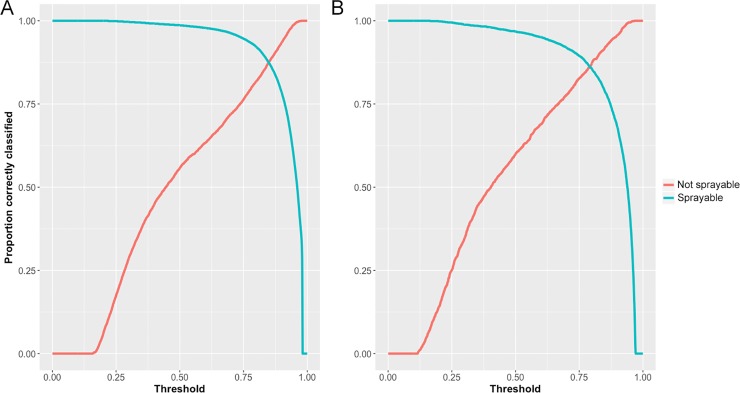
**Classification performance of the super learner model under different cutoff values for A–Botswana and B–Swaziland**.

Fig 4. **Influence of difference cutoffs on the ability to correctly classify sprayable and not-sprayable structures using the super learner model.** For Botswana, the cutoff value at which equally good performance was possible for classifying sprayable and not-sprayable structures was 0.85, leading to 87.4% of structures correctly classified in each class. The cutoff for the Swaziland model was 0.79, similarly leading to 86.2% of structures correctly classified in each class.

Using the test data, the super learner models achieved AUC values of 0.96 and 0.95 in Botswana and Swaziland respectively. Table 4 shows the confusion matrices generated using the pre-determined country specific cutoffs at which CV-classification accuracy was equal for sprayable and not-sprayable structures. In both countries, between 86.3%– 89.8% of sprayable and not-sprayable structures were correctly classified.

**Table 4:** Performance of the super learner models when applied to the test data. Classifications were made using the country specific cutoffs at which CV-classification accuracy was equal for sprayable and not-sprayable structures.

		Observed		
	Predicted		Not-sprayable	Sprayable
**Botswana**		Not-sprayable	468	458
		Sprayable	53	2937
		% correctly classified	89.8%	86.5%
	Predicted		Not-sprayable	Sprayable
**Swaziland**		Not-sprayable	167	100
		Sprayable	19	631
		% correctly classified	89.8%	86.3%

Predictions made using the final super learner model suggested that 63% and % of structures in Botswana and Swaziland, respectively, were sprayable (Table 5).

**Table 5:** Breakdown of predictions for all OSM structures in the country, including structures for which type is available.

Country	Sprayable	Not-sprayable
Botswana	385,336 (63%)	224,942 (37%)
Swaziland	378,659 (64%)	214,706 (36%)

## Discussion

While there has been a growth in the availability of data relating to the locations of man-made structures via projects such as OpenStreetMap, data relating to those structures are often limited. Yet, having information on the characteristics of buildings would increase the value these data have for a variety of purposes, including IRS for malaria. Despite its value, it is typically not known what the structure is used for, as this requires on the ground observation. This lack of granularity limits the value of these data. Here we show how the use of machine learning can be used to augment existing OSM data by predicting whether a building is residential, and therefore sprayable, or not. While the motivation of this study was to identify locations and numbers of residential buildings for planning malaria IRS campaigns, these data, and the underlying methods employed, may have utility across a number of sectors and fields.

While we restricted the base learners and predictors to those outlined in the methods, in theory it is possible to use any combination of classification base learners. The main limiting factor is computation time, although both the SuperLearner package and other desktop and cloud based platforms are becoming increasingly fast, taking advantage of parallel computing.

Similarly, there is, in theory, no restriction on the number of predictor variables that could be used. The spatially clustered nature of the type data meant that a spatial model, i.e. a model which incorporates the locations of the data, was not appropriate and may have led to spurious predictions. In situations where the data are more random in space, a spatial model may allow for improvements in predictions. This study also only included a crude estimate of structure shape, using number of sides. More sophisticated shape detection algorithms may boost performance but were beyond the scope of this study. An ongoing project predicting roof types may also prove valuable and will be included in future efforts.

It is not clear why there was a clear increase in performance with increasing urbanicity in Swaziland and not Botswana. This may be due to differences in the characteristics of buildings in very urban areas, or the fact that more training data were available in Botswana. Either way, AUC values were consistently above 0.9, suggesting very good discriminatory performance irrespective of setting.

While the study produced good results, there are several limitations and opportunities for further research. First, not all structures classified as sprayable from the OSM type field are actually sprayable. For example, a kitchen structure within a home may be classified as residential in OSM and yet is not typically sprayed. Conversely, in certain situations, structures such as schools are sprayed. Equally, there may be some error in the OSM classifications or bias could be introduced if building type information is only available for certain categories of structures, such as retail outlets or very large warehouses. Conducting a ground truth exercise was beyond the scope of this study, but would provide valuable information for future efforts.

Related, we also intentionally reduced the modeling problem to a binary outcome (sprayable and not-sprayable structures) limiting the usefulness of the data to other fields. Where multiple structure type classifications required, however, a similar ensemble approach could be employed. Indeed, ensemble modeling has been used with success for other multi-class classification problems [24, 25].

Third, it should be pointed out that this analysis is only really useful to programs in settings where every structure has been mapped or where the completeness can be quantified. If gaps exist of unknown size, as if often the case, estimates of numbers of sprayable structures will be low. While OSM does not currently have a mechanism to estimate the completeness of the building enumeration data, efforts are underway to provide this information.

Fourth, it was only possible to do this analysis as data on structure type were available for a reasonable subset of the data. For settings where this information is not available, or available for only a very small number of structures, it may be possible to use or pool data from neighbouring regions and extrapolate predictions. That said, efforts should be made to ensure the characteristics of structures in the prediction areas are similar to those used as training data. Alternatively, data on structure type could be collected for a subset of structures either on the ground or virtually via services such as Google Street View. As Google Street View is not available ubiquitously, being typically more widely available in urban areas, extrapolations should be made cautiously.

Fifth, in addition to estimates of the number of sprayable structures in a target area, an estimate of the wall surface area would enable more accurate estimate of the amount of insecticide required. While gold standard information on wall surface area was not available for Botswana and Swaziland, similar efforts have been conducted in Zambia, whereby building footprint was shown to correlate strongly with wall surface area [5]. Extrapolations from the Zambia study could therefore be used to provide a rough estimate in Botswana and Swaziland. Similarly, as it correlates strongly with wall type, the authors showed that an estimate of roof type is useful for determining the type of insecticide to be used. Unfortunately, gold standard information on roof type was not available in the two countries of focus here.

Here we have shown that machine learning can be used to augment the value of open source building data by predicting whether structures are residential or not. Future work will be focused on exploring alternative stacking methods as well as other predictor variables which could be used to boost performance of this modeling approach.
